# Plasma Protein Layer Concealment Protects *Streptococcus pyogenes* From Innate Immune Attack

**DOI:** 10.3389/fcimb.2021.633394

**Published:** 2021-05-20

**Authors:** Hilger Jagau, Swathi Packirisamy, Kyle Brandon, Heiko Herwald

**Affiliations:** ^1^Division of Infection Medicine, Department of Clinical Sciences, Lund, Lund University, Lund, Sweden; ^2^UCD School of Medicine, University College Dublin, Dublin, Ireland

**Keywords:** antibiotic resistance, antimicrobials, cold atmospheric plasma (CAP), hemostasis, innate immune system, *Streptococcus pyogenes* (GAS)

## Abstract

Early recognition and elimination of invading pathogens by the innate immune system, is one of the most efficient host defense mechanisms preventing the induction of systemic complications from infection. To this end the host can mobilize endogenous antimicrobials capable of killing the intruder by perforating the microbial cell wall. Here, we show that *Streptococcus pyogenes* can shield its outer surface with a layer of plasma proteins. This mechanism protects the bacteria from an otherwise lytic attack by LL-37 and extracellular histones, allowing the bacteria to adjust their gene regulation to an otherwise hostile environment.

## Highlights

The formation of a plasma protein layer around the bacterial surface protects streptococci from attack by host-derived antimicrobials.

## Introduction

In 2019 the *Interagency Coordination Group on Antimicrobial Resistance* (IACG) estimated drug-resistant bacterial infections account for more than 700,000 deaths globally (I.I.C.G.o.A. Resistance, 2019). According to the IACG this number will increase to 10 million in 2050, if no measures are taken and may force up to 24 million people into poverty (I.I.C.G.o.A. Resistance, 2019). Thus, it is not surprising that the IACG recommended an *One Health* approach to antimicrobial resistance, which is in line with the agenda 2030 sustainability goals (I.I.C.G.o.A. Resistance, 2019).

*Streptococcus pyogenes* is a Gram-positive, nearly-exclusively human pathogen causing an estimated 600 million infections per year (Carapetis et al., 2005) of which more than 18 million are serious (Ralph and Carapetis, 2013). Notably, evidence is emerging that *S. pyogenes* can develop resistances to antibiotics such as tetracycline (Sanson et al., 2019), β-lactam antibiotics (Yu et al., 2020), and macrolides (Sanson et al., 2019). Considering that *S. pyogenes* is found in the list of the top ten causes of mortality from infectious diseases (Ralph and Carapetis, 2013), the occurrence of antibiotic resistances bears the risk of a worldwide increase of severe streptococcal infections.

In order to colonize and proliferate within the invaded host, *S. pyogenes* expresses a panel of surface-bound and secreted proteins that can help the bacterium to adapt to its hostile environment and/or cause pathologic immune responses. One family of surface-bound proteins, also referred to as M proteins or M-like proteins (Frost et al., 2018) have been found to mediate contact to cellular surfaces (Ochel et al., 2014) and to interact with a number of plasma proteins such fibrinogen (Herwald et al., 2004), immunoglobulins (Berge et al., 1997) and albumin (Akesson et al., 1994). Using a mass spectroscopy-based approach more than 180 plasma proteins were found to interact with the surface of *S. pyogenes* bacteria (Sjoholm et al., 2014). In the same study it was also found that the interaction with plasma proteins was to a great deal mediated by M proteins and M-like proteins (Sjoholm et al., 2014).

The present study was undertaken to understand how *S. pyogenes* adapts to an attack by endogenous and exogenous antimicrobial substances. Our results show that upon contact with human plasma the bacteria surround themselves with a layer made up by host proteins. The formed shell not only protracts the direct interaction of the bactericidal reagents with the bacterial cell wall, but it also gives the bacteria the chance to use the gained time to up-regulate virulence factors and proteins for better survival in a hostile environment. Our results also suggest that M- and M-like proteins play an important role in forming the proteinous shield around the bacterial surface.

## Material and Methods

### Bacterial Cultivation

The *S. pyogenes* serotypes AP1, AP4, and AP12 were obtained from the WHO collaborating center for Reference and Research on Streptococci, Prague, Czech Republic. *S. pneumoniae* F23 was obtained from the Gothenburg Cooperation Lab and *E. coli* K12 from the Institute of Microbiology, Technical University of Braunschweig, Germany. For the experiments with *S. aureus* bacteria the strain Newman was used which is a coagulase- and clumping factor-positive strain (Boden and Flock, 1989). AP1 M-protein mutant MC25, with a non-covalently bound M1 protein was kindly provided by Prof. Mattias Collin, Department of Clinical Sciences, Lund Division of Infection Medicine, Lund University, Sweden. It should be noted that this strain lacks the ability to convert SpeB, a secreted streptococcal cysteine proteinase, into its enzymatically active form (Kihlberg et al., 1995). Because this strain does not generate a protein layer around the bacterial wall, this strain was not used when testing the effect of antimicrobials. All bacteria, with the exception of the *E. coli* K12 strain, were first cultured overnight on blood agar plates from glycerol stocks stored in -80°C cryotubes. The *E. coli* K12 strain was cultured on THY plates. For cultivation of MC25, kanamycin (75µg/ml) was plated directly on the blood agar plates and later added to the THY media in the liquid culture. All bacteria were then cultured in 10ml Todd-Hewitt broth supplemented with yeast extract (30g/l Todd-Hewitt broth, 10g/l yeast extract) at 37°C in a 5% CO_2_ in 15ml cultivation screwable tubes.

### Human Plasma Preincubation

Starting culture in THY-media was grown till OD_600 =_ 0.1 and then split and refilled with new media to enable equal growth of cultures to late exponential phase of OD_600_ of 0.45. Bacteria were gently centrifuged at 1.000 x rcf for 10min and washed twice with 1 x PBS. After the last resuspending step, the OD_600_ was adjusted to a starting CFU-count around 1 x 10^5^ bacteria/ml and centrifuged again. This time bacterial pellets were resuspended in assay buffer (150mM NaCl, 20mM Tris, pH 7.6) and then equally incubated in the same volume of 10% thawed prewarmed human plasma or in assay buffer. Tubes were placed horizontal in the incubator for 5 or 30min at 37°C, 5% CO_2_. Bacteria were again gently centrifuged at 1.000 x rcf for 10min, washed twice with assay buffer and then used for further assays. Assay buffer-incubated bacteria were used in all experiments as negative controls. For the killing assays in solution 100µl of the bacterial solution, either buffer or plasma incubated, were mixed with 100µl of the testing solution.

### Human Plasma Samples

Human plasma derived from human peripheral blood, was collected from healthy volunteers (IRB approved protocol Dnr 2017/728) in a 2.7ml vacutainer containing citrate (0.3ml 0.109 M sodium citrate, BD Vacutainer system). Vacutainer tubes with collected blood were inverted carefully to mix blood and anticoagulant. Samples were centrifugated immediately for 10min at 1300 x rcf, at room temperature. Plasma was aliquoted and frozen at -80°C and thawed for a maximum of two times.

### Preparation of TEM Samples for Buffer/Plasma Incubation

For the transmission electron microscopy (TEM), bacterial solution (200µl), was incubated for different timepoints (5, 30, 60 and 120min). The samples were immediately mixed with 1ml EM-fix (2.5% glutaraldehyde in 0.2M Na-cacodylate buffer, pH 7.2) to preserve the formed surface structures. Samples were then left at room temperature for 24h and further processed for TEM. Embedding and negative staining was done by IQ-Platform, Lund University.

### Cold Atmospheric Plasma Treated Buffer

Assay buffer (500µl) was treated with cold atmospheric plasma (CAP) with the KINPen^®^ MED (Settings:30s, 2.5bar, 3l/min) in a 6-well plate. The KINPen^®^ MED was kept at a stable distance throughout. This distance was based on the length of an adapter for skin treatment added by the supplier. The CAP-buffer was used 5-10min after preparation. The bacteria were resuspended and incubated (5min) with CAP buffer (500µl) after washing. The bacterial survival assay was performed as mentioned below.

### Bacterial Survival Assay

After the washing step, bacteria were resuspended in assay buffer. CAP incubation was followed by LL-37, calf thymus histones (CTH) or tetracycline incubation. Bacteria were resuspended in CAP-treated buffer as described above before adding other solutions. Final concentrations of CTH (0.1μg/ml), LL-37 (10μM) and tetracycline (30μg/ml) were added all in assay buffer. CTH was purchased from Merck Millipore (Darmstadt, Germany) and LL-37 trifluoroacetate salt (human) was purchased from Schafer-N (Copenhagen, Denmark) and both stored at -20°C. Tetracycline hydrochloride was purchased from Thermo Fisher and stored at 4°C in the dark. After the incubation time ended (30 or 60min), 100µl were taken from the reaction mixture and serial dilutions were performed in a 96-well plate followed by plating on THY plates in triplicates.

### Fibrin Clot Assay

After preincubation and washing, bacterial solutions were mixed with fibrinogen in solution on a coverslip. Fibrin network formation was initiated by adding human thrombin (Merck Millipore). 25µl of the bacterial solution was mixed with 25µl of the fibrinogen stock solution (100mg/1700µl in sterile water, prewarmed for at least 24h at 37°C). The mixture was then added to rat tail collagen I coated coverslips (final concentration: 50µg/ml diluted with 0.01M HCl). The clot was gently resuspended with 200µl assay-buffer, containing testing substances. The covered clot was then incubated for 30 or 60min at 37°C, in a 5% CO_2_ atmosphere. CLEM analysis involved the same procedure with the inclusion of indium titandioxid cover glasses coated with collagen I. Samples were prepared for CLEM by the IQ-Platform, Lund University.

### Live/Dead Staining and Microscopical Analysis

LIVE/DEAD™ staining (LIVE/DEAD™ BacLight™ Bacterial Viability Kit from Thermo Fisher) was used to identify bacterial cells which have structural integrity deficiency in their cell wall enabling propidium iodide staining. Propidium iodide red stained cells were considered as dead. For a LIVE/DEAD™ staining the solution in the well was removed after the incubation time of 30min or 60min and 200µl assay buffer with 1/1 mixture of component A and B (total vol. of the mixture 1µl each) were added and incubated for 30min at 37°C, protected from light. Afterwards the wells where carefully washed three times with 300µl of fresh assay buffer and then incubated overnight in 4% PFA solution at room temperature to preserve the sample. All cells where stained by the DNA intercalating dye SYTO 9. Using a Nikon microscope (TI2 body) and an Andor Zyla 4.2 sCMOS camera, 10 z-stacks with a thickness of 3µm were taken from representative areas per coverslip.

### Image J Based Analysis

Merged z-stacks images where further semi-automatic processed with ImageJ (1.52p 64-bit) using the find-maxima-function for red and the green fluorescence image analysis. For this purpose, images were converted to grey scale. For counting the prominence for maxima was set to 3 up to 4 for the TRIC-channel and to more than 10 for the FITC-channel. Counting was examined manually by zooming in and review counted signals by eye. Percentage killing was calculated by subtracting counts between the two channels, green (all) and red (dead/membrane ruptured) cells. For a better comparison of effects from LL-37 and CTH a background normalization was done for the killing observed after 30min and 60min of the only buffer and plasma incubated bacteria.

### RNA-Purification and q-PCR With Applied Fold Change Method

The RiboPure™-Bacteria Kit was used for the RNA-extraction. Briefly, bacteria were incubated as described before for the bacterial survival assay. After 0, 30 and 60min, samples were centrifuged at 1.000 x rcf for 10min and resuspend in RNAwiz and transferred to the kit tubes with Zirconia Beads. Samples were processed according to the Kit protocol. Extracted nucleic acids were measured with the NanoDrop™ 2000 spectrophotometer and genomic DNA was first eliminated before converting the remaining extracted RNA to cDNA. Both steps were done with the QuantiTect Reverse Transcription Kit, purchased from Qiagen (Hilden, Germany). Afterwards cDNA amount and quality were determined using the NanoDrop™ 2000 spectrophotometer. 300ng was used as a template for each Q-PCR master mix. Expression of virulence genes, M-protein, SpeB, HasB and DNA gyrase, Sortase A, FhuB, and HtrA of AP1 were determined. A modified PCR master mix was prepared, following the standard Thermo Fisher protocol (**Table S1**). The following thermal cycling conditions were used in a QuantStudio™ 7 Flex Real-Time PCR Instrument: UNG incubation for 2min at 50°C, polymerase reaction for 10min at 92°C and denatured at 95°C for 1min with an annealing/extending time of 20sec at 60°C. The primers and probes (**Table 1**) were designed based on published target sequences and by using the NCBI primer BLAST program along with the online qPCR Primer & Probe Design Tool from Eurofins (von Pawel-Rammingen et al., 2002). Expression fold change (EFC) was calculated by using the 2-ΔΔCt method (Akesson et al., 1996). DNA gyrase, a known housekeeping gene (Frick et al., 2011), was chosen as the housekeeping gene.

**Table 1:** Streptococcal gens for RT-qPCR analysis.

Gen	Shortening	Function	Used for evaluating	Ref.
DNA gyrase subunit A (SPy_1152)	DNAgy	A topoisomerase that negatively supercoils closed circular double-stranded DNA (ds)*	Housekeeping/adjust gene expression	(Goerke et al., 2001; Pappesch et al., 2017)
Ferrichrome ABC transporter (Spy_0385)	FhuB	A ferrichrome ABC transporter	Iron uptake/Virulence	(Lei et al., 2003)
Hyaluronan synthase HasB (Spy_2200)	HasB	A protein involved glycosaminoglycan synthesis	Virulence	(DeAngelis et al., 1994)
M-protein (Spy_2018)	M-pro	A surface anchored virulence factor	Virulence	(Akesson et al., 1994)
Serine protease HtrA (SPy_2216)	HtrA	A chaperon involved in cleaving the SpeB zymogen	Surface chaperon activity	(Poquet et al., 2000)
Streptococcal cysteine protease streptopain (SPy_2039)	SpeB	A streptococcal virulence factor cleaving human fibronectin and degrades vitronectin*	Virulence	(Kortt et al., 1976)
Transpeptidases Sortase A (Spy_1154)	SortA	A protein involved in anchoring streptococcal surface proteins	Protein cargo	(Race et al., 2009)

*www.uniprot.org.

### Statistical Analysis

Statistical analyses were performed using Prism (8.3.0). The data were first tested for normal distribution. In case not all data sets followed a Gaussian distribution a Mann-Whitney-test was performed to evaluated, if there were significant differences between treatments. For the clot assay the buffer and plasma incubation with N=18 a Dunn’s multiple comparison was performed and for the LL-37 and CTH clot assays a Mann-Whitney test was used. In all other cases a one-way ANOVA (Tukey’s multiple comparisons test) was chosen. Rt-qPCR-experimental data were analyzed using a non-parametric Student’s t-test for significance, with no significance detected. The pre-formed clot experiments were done as biological duplicates with each completed in technical duplicate. All other experiments were based on technical quadruplicates and biological triplicates.

## Results

### Interaction of Human Plasma With Different Bacterial Species

**Figure 1:**
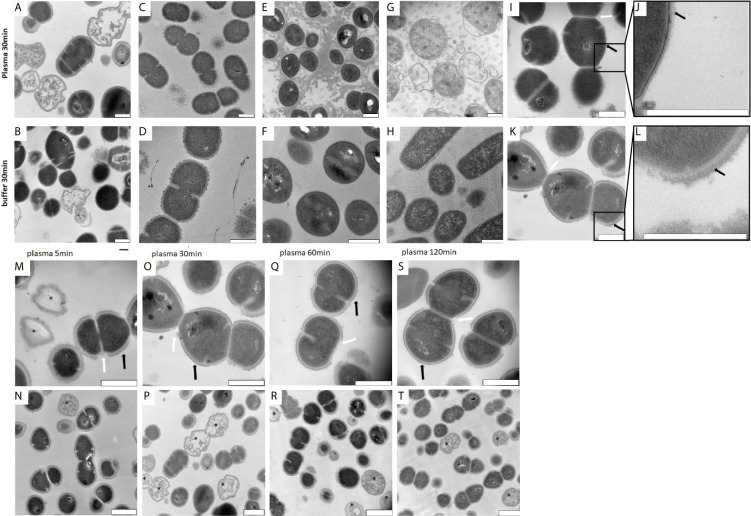
**(A–H)** TEM images of *S. pyogenes*, *S. aureus*, *E. coli* and *S. pneumoniae* after incubation with buffer or plasma. Bacteria were incubated with buffer or plasma as described in the *Material and Methods* section. Images were taken of thin sectioned *S. pyogenes* **(A, B)**, *S. pneumoniae* **(C, D)**, *S. aureus* **(E, F)** and *E. coli* **(G, H)**. The precipitated proteins seen in **(E)** appearing as a cloud, are most likely generated upon contact of plasma with staphylococcal clumping factors. Scale bars 500nm. **(I–L)**. Surface comparison of *S. pyogenes* AP1 bacteria before and after incubation with human plasma. *S. pyogenes* AP1 bacteria were grown to exponential phase and incubated with buffer **(I, J)** or human plasma **(K, L)** as described in *Material and Methods*. Black arrows point towards proteins on the cell wall surface. White arrows point at gaps where septum formation occurs. Scale bars 500nm. **(M–T)** TEM time kinetic series of plasma incubated *S. pyogenes* bacteria. The *S. pyogenes* AP1 strain was grown to exponential phase and incubated with 5% human plasma in assay buffer for 5min **(M, N)**, 30min **(O, P)**, 60min **(Q, R)**, and 120min **(S, T)** as described in *Material and Methods*. After a washing step bacteria were subjected to TEM. Black arrows point towards plasma shell structures on the cell wall surface and white arrowheads to the gaps in the shell at bacterial regions where septum formation occurs. Scale bars 500nm **(M, O, Q, S)** and 1µm **(N, P, R ,T)**. Dead bacteria are marked with *.

Previous work has shown that upon contact with plasma, group A streptococci are covered with a proteinous layer around their outer surface (Naudin et al., 2015). To test whether the formation of the proteinous layer is part of an immune response or a species-specific bacterial escape mechanism, we incubated human plasma with *Streptococcus pneumoniae*, *Staphylococcus aureus*, or *Escherichia coli* bacteria and analyzed whether similar shell formation can be detected as seen with *S. pyogenes* bacteria. To this end, the four species were treated with plasma for a short (5min) or a longer (30min) incubation time. This was followed by a washing step and subsequent examination by transmission electron microscopy (TEM). **Figures 1A–H** shows that only *S. pyogenes* bacteria are able to surround themselves with a protein layer, while all other species lack this ability. These results suggest that the formation of the layer is driven by *S. pyogenes* rather than host immune response.

In the next series of experiments, we explored the proteinous shell morphology of *S. pyogenes* AP1 bacteria after their incubation with human plasma. In line with previous observations, we found in the absence of plasma, the bacteria are covered with hair-like fibrils built up by M1 protein and protein H (Collin and Olsen, 2000; Herwald et al., 2003) (**Figures 1I–L**). When incubated with human plasma, the newly formed protein cover was attached to the extension of the hair-like structures, suggesting that the layer is bound to the outer bacterial surface proteins with a thickness between 40nm and 80nm (**Figures 1K, L**).

**Figure 2:**
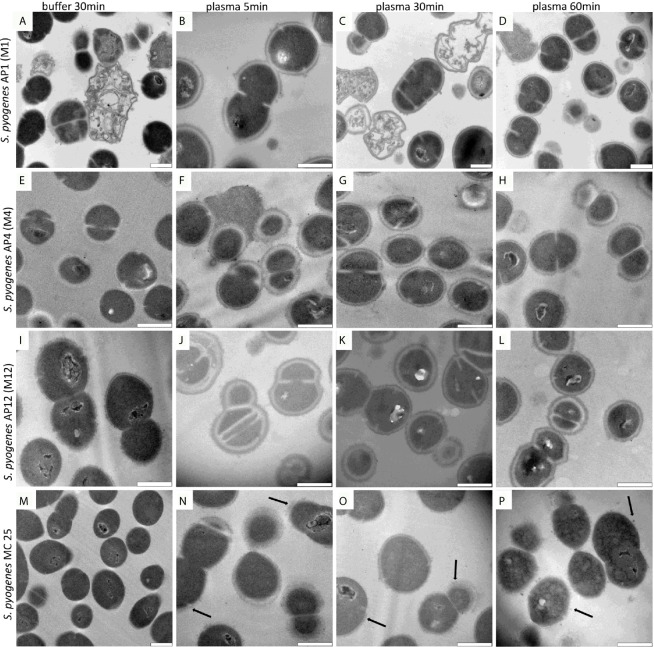
**(A–P)** AP1,4,12 and MC25 TEM time lapse series after plasma incubation. The *S. pyogenes* strains were grown to exponential phase and incubated in 5% human plasma in assay buffer for 5min **(B, F, J, N)**, 30min **(C, G, K, O)**, 60min **(D, H, L, P)**, and in buffer for 30min **(A, E, I, M)** as described in *Material and Meth*ods. Thereafter bacteria were washed and subjected to TEM. Black arrows point to partial bound plasma proteins directly at the surface of MC25. Scale bars 500nm.

When performing a time kinetic study we found that *S. pyogenes* AP1 bacteria were already covered 5 minutes after being added to human plasma (**Figures 1M, N**). No major changes of thickness of the proteinous layer were seen upon longer incubation times (**Figures 1M, O, Q, S** black arrows), except that the diameter size decreased at some septum formation sites (**Figures 1M, O, Q, S** white arrows). At lower magnification (**Figures 1N–T**) the images also show that some bacteria have lost their cytosolic content (**Figures 1M, N, P, R, T** marked with a black star), suggesting that not all bacteria can survive within the plasma protein shell. Subjecting the *S. pyogenes* strains AP4 (an M4 serotype, **Figures 2E–H**) and AP12 (an M12 serotype, **Figures 2I–L**) to the same experimental conditions, a similar shell formation was noted, though less dead bacteria were seen. An isogenic AP1 mutant strain (MC25) was employed as a control, that lacks surface-bound M1 protein (Kihlberg et al., 1995). As depicted in **Figures 2M–P**, plasma proteins did not completely cover the surface of the MC25 strain and a gap formation was not noted as seen for the other streptococcal strains. Also the protein layer did not completely cover the bacterial surface of the MC25 strain (**Figures 2N–P** black arrows).

### The Protein Shell Promotes Resistance to Bacterial Killing

**Figure 3:**
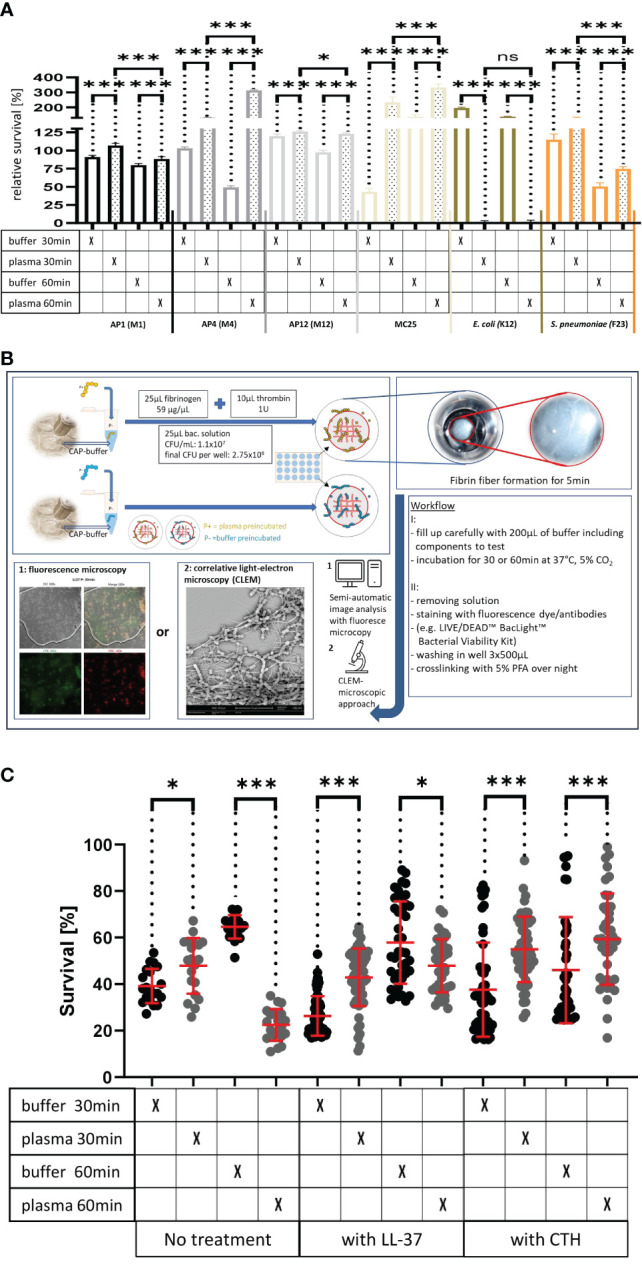
**(A)** Survival of the streptococcal strains AP1, AP4, AP12, MC25, *E. coli* K12 and *S. pneumoniae* F23 following incubation with buffer or plasma. The bacterial strains were grown to exponential phase and incubated in 5% human plasma and assay buffer for 30min. Bacteria were then washed and incubated for another 30min or 60min. The graph represents the relative survival rate, calculated based on the platings for the 30min and 60min time points divided through the starting CFU before the plasma and buffer incubation. Except for K12. Instead of the starting CFU after the plasma incubation, the CFU after buffer incubation was chosen. This was done since the buffer CFU differs from 112.6x10^4^ after buffer incubation to 2.6x10^4^ after plasma incubation and would mislead to a high positive survival rate by increasing to 5.7 x10^4^ at 30min. CFU counts were measured from 10^-4^ dilutions on THY agar plates. Assays were performed as technical quadruplicates with biological triplicates. Significance bars: To compare buffer and plasma sample values at the same time point a one-way ANOVA (Tukey’s multiple comparisons test) was used. *p ≤ 0.05, ***p ≤ 0.001, ns p=not significant. **(B)** Workflow with in vitro fibrin clot assay. Upper left starting with resuspending the buffer and plasma incubated bacteria in normal or CAP-treated buffer. Afterwards clot assay was started by mixing bacterial solution as well as fibrinogen and thrombin on the collagen coated coverslip. Initial fibrin network formation was enabled for 5min before bacterial clot was floated with buffer including components to test. 30min and 60min time points were further processed and either used for LIVE/DEAD™ BacLight™ staining or processed for correlative light-electron microscopy (CLEM). For the CLEM only scanning electron microscopy (SEM) was used to visualize fibrin network formation. **(C)** LL-37 and CTH induced bacterial killing inside a fibrin clot. S. pyogenes AP1 bacteria were grown to the exponential phase, incubated with 5% human plasma (grey dots) or buffer (blackdots), and then embedded in a fibrin network. After a 30min and 60min incubation time bacterial survival was measured inside the clot. For buffer/plasma without N=18; plasma 30min CTH N=55; buffer 30min CTH N=49; plasma 60min CTH N=42; buffer 60min CTH, buffer 30min LL-37, buffer 60min LL-37, plasma 60min LL-37 all N= 40; plasma 30min LL-37N=59 and for buffer 30min LL-37 N=60. The graphs display meanand standard error. Statistic tests performed on Prism (8.3.0) using aMann-Whitney test. *p ≤ 0.05, ***p ≤ 0.001.

The TEM results suggest that streptococci of the AP1 strain (**Figures 2A–D**) are more susceptible to bacterial killing, when embedded in the plasma protein shell compared to the other streptococcal strains tested. This observation prompted us to examine whether shell formation can influence bacterial viability of streptococcal strains AP1, AP4, AP12, and MC25, respectively. **Figure 3A** shows that all S. pyogenes wild type strains tested were able to grow and proliferate when surrounded with a plasma protein layer (with the exception of the AP1 strain where survival after 60min was decreased compared to 30min). In contrast, the isogeneic AP1 mutant MC25, lacking surface-bound M-protein on its surface, was not able to form a layer that covered the entire bacterial surface (**Figures 2M–P**). Though the growth rates were for all strains statistically significant (**Figure 3A**), bacterial proliferation and the relative survival (**Figure 3A**) were considerably lower for the AP1 strain compared to the other strains, which is in line with the findings of the TEM experiments (**Figures 2A–P**). *E. coli* K12, a non-pathogenic Gram-negative enterobacterium was significant eradicated after the plasma preincubation compared with the buffer incubation. The *S. pneumoniae* strain F23, which was isolated from a meningitis patient in Gothenburg showed similar growth behavior after the plasma preincubation as AP4 and AP12.

Previous results have shown that activation of the coagulation system and subsequent formation of a fibrin network not only crosslinks the bacteria within a formed fibrin clot (Loof et al., 2011), but also leads to the generation of fibrinogen-derived antimicrobial activity (Pahlman et al., 2013). Both findings emphasize the importance of the coagulation system in the early innate immune system. Considering this role, the next experiments were set up to study whether the formation of the plasma protein shell around the streptococcal surface is a bacterial defense mechanism that can rescue immobilized streptococci of the AP1 strain from an otherwise lethal immune attack. To prove this hypothesis bacteria were preincubated in the presence or absence of human plasma. Bacteria were then added to a fibrinogen solution directly on a collagen I coated coverslip. The mixture was then treated with thrombin to allow the generation of a fibrin network for 5min before the clot was covered with buffer including antimicrobials (see also workflow shown in **Figure 3B**).

After 30min and 60min incubation bacterial survival was microscopically determined using a LIVE/DEAD™ staining kit. **Figure 3C** depicts that under these experimental conditions bacterial survival is enhanced for the 30min, but decreased at the 60min time point, when AP1 bacteria were preincubated with plasma. **Figure 3C** also show that inside a fibrin clot, the plasma shield prevents the streptococci from killing by LL-37 or CTH. For LL-37 at the 60min timepoint, more bacteria survived after buffer pre-incubation than after the plasma incubation (**Figure 3C**). CTH-treated samples had at both time points significantly increased survival for the plasma incubated samples (**Figure 3C**).

### The Protein Shell Delays Killing From Innate Immunity Antimicrobials

**Figure 4:**
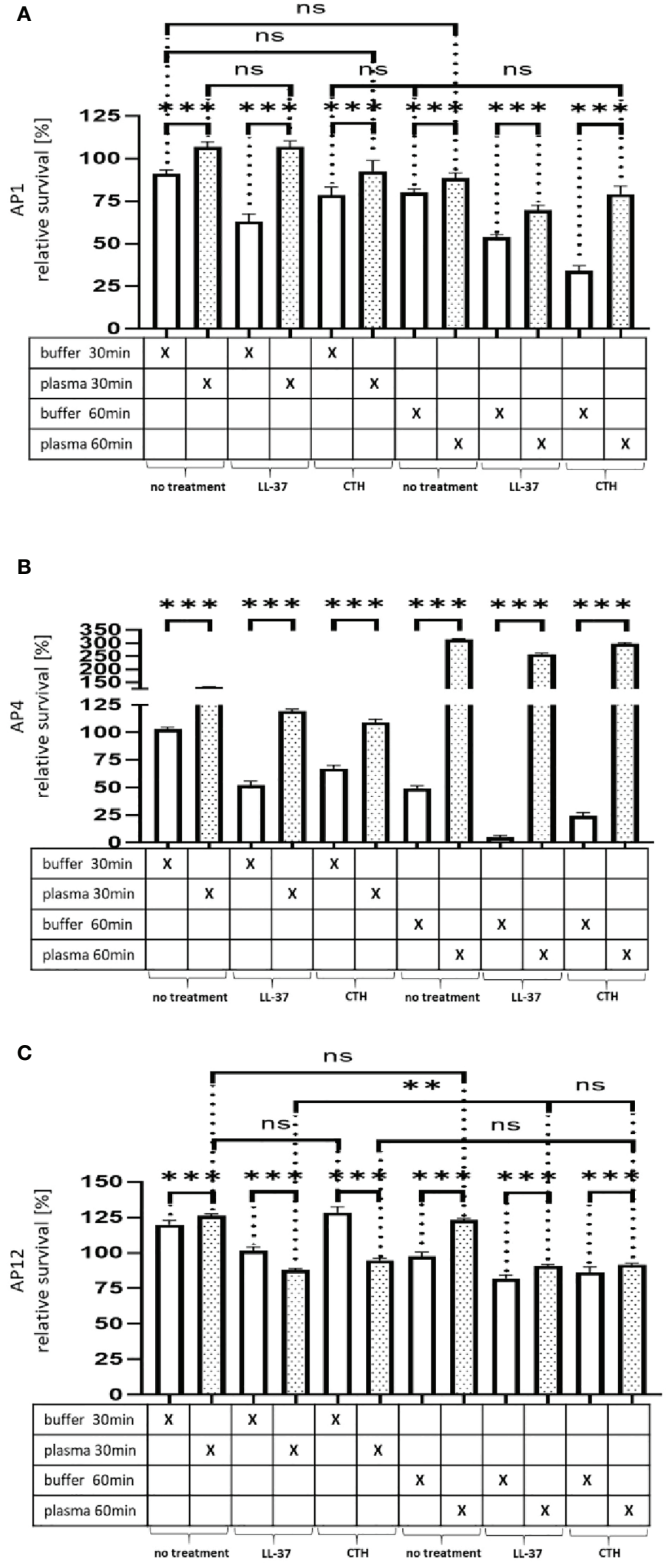
Survival of the streptococcal strains AP1, AP4 and AP12 upon treatment with LL-37 and CTH. The bacterial strains AP1, AP4 and AP12 were grown to exponential phase before adding to 5% human plasma or assay buffer for 30min. After a washing step the bacteria were incubated for another 30min and 60min in the presence of LL-37 and CTH **(A–C)**. Incubation with buffer served as control. The graph represents the relative survival rate, calculated based on the platings for the 30min and 60min time points divided through the starting CFU before the plasma and buffer incubation. CFU counts were measured from 10^-4^ dilutions on THY agar plates. The graphs display mean and standard error. Statistic tests performed on Prism (8.3.0) using a one-way ANOVA (Tukey’s multiple comparisons test) to compare buffer and plasma sample values at the same time point and to compare similar conditions over two timepoints. ***p ≤ 0.001, **p ≤ 0.01 and ns p=not significant. Significance was calculated based on technical quadruplicates per individual biological sample with performing the assay three times independently.

Having shown that streptococci can proliferate and escape the host immune response, when covered with a plasma protein layer, we next tested whether the formed shell will also protect the bacteria against other antimicrobial peptides. To this end, the three strains (AP1, AP4, and AP12) were incubated with buffer or human plasma for 30min and exposed for 30min and 60min to sublethal doses of LL-37 and calf thymus histones (CTH). As shown in **Figures 4A–C** the treatment with LL-37 led to a decrease in survival of all three strains, when the bacteria were buffer preincubated, while the preincubation with human plasma made the bacteria more resistant to the LL-37 attack with exception of the 30min time point for AP12. The protective effect was most significant in the streptococcal AP4 strain (**Figure 4B**), whereas the differences in survival of the streptococcal AP12 strain was, though statistically significant, less apparent (**Figure 4C**).

Like LL-37, also histones are considered as endogenous antimicrobial peptides. They are released for example from necrotic cells and have been shown to directly kill bacteria, fungi, and other parasites (Hoeksema et al., 2016). As depicted in **Figure 4A–C**, when CTH were tested under the same experimental conditions, similar findings were obtained as seen for LL-37. Together these data show that the shield formed by plasma proteins protects the bacteria from an endogenous antibacterial immune response.

### Interference of the Plasma Protein Shield With Tetracycline and Cold Atmospheric Plasma Treatment

Our previous results revealed that the plasma protein shield protects streptococci from an innate immune attack caused by endogenous antimicrobials. In the next series of experiments, we examined whether the activity of exogenous antimicrobial substances can additionally be abolished by the same mechanism. Notably, penicillin is still the drug of choice to treat *S. pyogenes* infections in hospitals. However, in cases of allergic reactions towards penicillin, antibiotics such as tetracycline, macrolides, and lincosamides are used as replacement therapy. While streptococcal resistances towards penicillin do not constitute a clinical problem, resistances to tetracycline and the other mentioned antibiotics have started to emerge (Sanson et al., 2019). Considering this aspect, we decided to focus on tetracycline throughout the remaining part of this study. Notably, tetracycline is below 0.5 kDa in size which may allow the antibiotic to penetrate the shielding layer. Thus, once it has passed the plasma layer and has been translocated inside the bacterium, the antibiotic should interfere with ribosomal translation processes.

**Figure 5:**
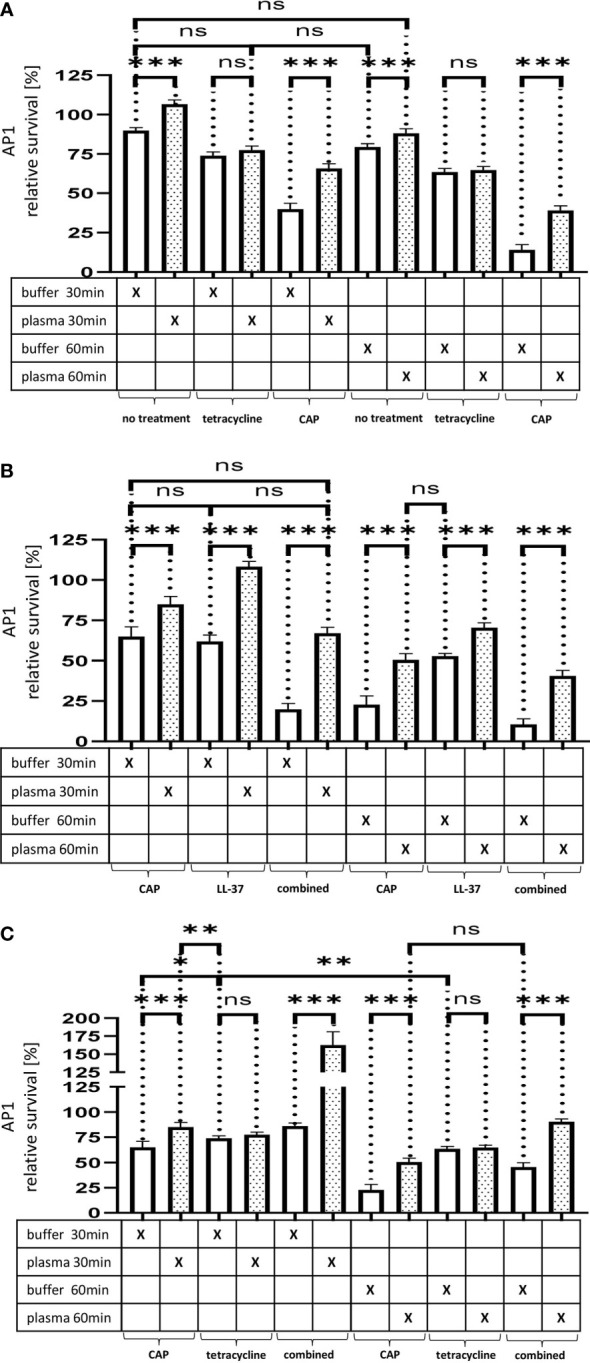
**(A)** The effect of tetracycline and cold atmospheric plasma on *S. pyogenes* bacteria covered in a plasma protein layer. *S. pyogenes* AP1 bacteria were grown to the exponential phase and incubated with 5% human plasma or buffer. The bacteria were then incubated with tetracycline or in CAP-treated buffer for 30min and 60min to assay buffer. The graph represents the relative survival rate, calculated based on the platings for the 30min and 60min time points divided through the starting CFU before the plasma and buffer incubation. CFU counts were measured from 10^-4^ dilutions on THY agar plates. The graphs display mean and standard error for the 30min and 60min timepoint. Significance was calculated using Prism (8.3.0) performing one-way ANOVA (Tukey’s multiple comparisons test) based on technical quadruplicates per individual biological sample with performing the assay three times independently. ***p ≤ 0.001, ns p=not significant. Survival of *S. pyogenes* bacteria upon combined treatment with CAP and LL-37 or CAP and tetracycline. S. pyogenes AP1 bacteria were grown to the exponential phase and incubated with 5% human plasma or buffer. The bacteria were then subjected to CAP-treated buffer and added for 30min and 60min to a buffer containing LL-37 **(B)** or tetracycline **(C)**. The graph represents the relative survival rate, calculated based on the platings for the 30min and 60min time points divided through the starting CFU before the plasma and buffer incubation. CFU counts were measured from 10^-4^ dilutions on THY agar plates. The graphs display mean and standard error for the 30min and 60min time point. Significance was calculated using Prism (8.3.0) performing a one-way ANOVA (Tukey’s multiple comparisons test) based on technical quadruplicates per individual biological sample with performing the assay three times independently. * p ≤ 0.033, ** p ≤ 0.01, *** p ≤ 0.001, ns p=not significant.

As depicted in **Figure 5A**, our experiments show that in contrast to LL-37 and CTH the plasma protein shield was not able to block the effect of sublethal doses of tetracycline. Thus, no significant differences in streptococcal proliferation were noted under these experimental conditions. In search for alternatives to traditional antibiotic treatment, cold atmospheric plasma (CAP) has recently attracted considerable attention (Brany et al., 2020). CAP is considered the fourth state of matter apart from solids, liquids, and gases. It is generated by applying an electric field to inert gases, typically helium or argon (Heinlin et al., 2011). During this process the gas becomes ionized and can build reactive radicals with a broad antibacterial activity (Brany et al., 2020). When applying sublethal doses of CAP-treated buffer instead of tetracycline to the bacteria, streptococcal survival was significantly decreased after 30min and even further decreased after 60 min (**Figure 5A**). At both time points survival was increased when the bacteria were covered with a plasma protein layer, suggesting that the shield caused a delay in antimicrobial activity of CAP-treated plasma.

When using a combined approach starting with a CAP treatment followed by the addition of LL-37 or tetracycline, we observed that CAP modified the two antimicrobials differently. **Figure 5B** show that a combined CAP and LL-37 treatment significantly decreased bacterial survival. On the other hand, when a combined CAP and tetracycline treatment was employed, bacteria survival increased, suggesting that CAP limited tetracycline antimicrobial activity (**Figure 5C**).

### Shield Formation Alters Streptococcal Virulence Gene Expression Upon Treatment With Tetracycline

**Figure 6:**
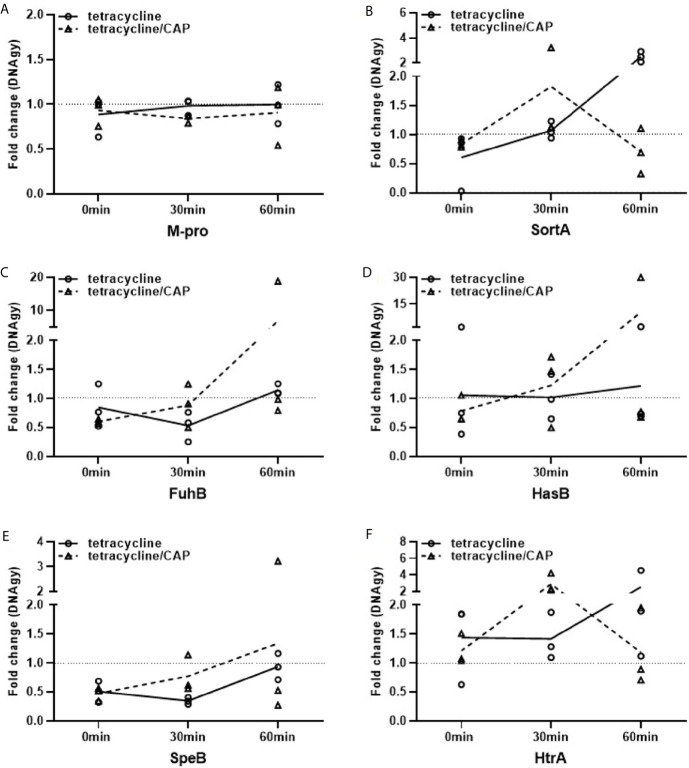
Regulation of virulence and metabolic genes of streptococci upon plasma protein layer coverage. Expression fold changes (EFCs) of virulence genes M-protein **(A)**, Sortase A **(B)**, FhuB **(C)**, HasB **(D)**, SpeB **(E)** and HtrA **(F)** of AP1. EFCs were calculated based on Ct-values of the selected genes after 0, 30 and 60min. Streptococci of the AP1 serotype were either treated with tetracycline or a combination of cold atmospheric plasma (CAP) and tetracycline. EFCs were adjusted against mean expression of DNAgy. Back dotted line is placed on EFCs of 1 as border for up- and down regulation of gene expression.

Our previous results demonstrated that plasma protein shield formation allows the bacteria to proliferate in an otherwise hostile environment. These findings prompted us to investigate whether the gene regulation of six selected streptococcal virulence factors or proteins involved in metabolism are affected when bacteria were treated with tetracycline, CAP or in combination with both (**Table 1**). As tetracycline inhibits bacterial proteins synthesis by blocking the interaction between aminoacyl-tRNA with the bacterial ribosome (Chopra and Roberts, 2001), the transcription of genes should not be affected. Subsequent RT-qPCR show that the gene-regulation of M1 protein was not influenced when the bacteria were incubated with plasma followed by a treatment with tetracycline or a combination with tetracycline and CAP (**Figure 6A**). Other virulence factors, however, such as the hyaluronan synthase (HasB) and the streptococcal cysteine proteinase (SpeB) were found upregulated, especially when applied in combination (**Figures 6D, E**). Furthermore, an upregulation of the ferrichrome ABC transporter (FhuB, **Figure 6C**) was noted, while the genes for the transpeptidases sortase A (SortA) and the putative serine protease HtrA were only up regulated when treated with tetracycline. When a combined treatment was applied, both genes were initially (after 30min) upregulated followed by a downregulation (60min) as depicted in **Figures 6B, F**. Together these data show that different antimicrobial treatments trigger distinct gene regulation within the proteinous layer, though more analysis is required to unravel the molecular mechanisms more precisely.

## Discussion

The coagulation system of vertebrates is evolutionarily conserved, evolving more than 400 million years ago (Davidson et al., 2003). It is one of the fastest responses that is triggered during wound healing. Activation of coagulation prevents the efflux of blood components into the surrounding tissue, whilst simultaneously acting as the first line of defense by inhibiting the entry of microbial pathogens into the blood stream (Loof et al., 2011). Additionally, the coagulation system makes use of a number of different defense mechanisms, such as bacterial entrapment in fibrin clots that prevents the systemic dissemination of the microorganism in the blood circulation (Wang et al., 2010). Other innate immune functions of the coagulation system include the induction of antimicrobial peptides by limited proteolysis of coagulation factors (Papareddy et al., 2010; Pahlman et al., 2013; Papareddy et al., 2018). Evolution has enabled microbial pathogens to adjust to host defense mechanisms by creating niches where the microorganisms not only survive, but also proliferate and challenge the invaded host (Thomer et al., 2016). Bacteria of the species *S. pyogenes* have also developed mechanisms that help to counteract their eradiation by the innate immune system. These mechanisms include IdeS, an IgG-cleaving and neutralizing protease (von Pawel-Rammingen et al., 2002) and protein SIC, a protein that can shield the bacterium from a complement attack (Akesson et al., 1996) and is able to inactivate antimicrobial peptides (Frick et al., 2011). Other enzymes such as streptococcal lysins, i.e. streptolysin O and streptolysin S, have been described to impair neutrophil oxidative burst and antibacterial responses (Uchiyama et al., 2015) and promote paracellular invasion of the bacteria across the epithelial barrier to Group A Streptococcus (Sumitomo et al., 2011), respectively. In this study we present another and so far undiscovered mechanism.

We show that bacteria of the *S. pyogenes* serotypes M1, M4, and M12 cover themselves with a layer of host proteins, that is generated within minutes after their contact with human plasma. This formed shell does not only provide short term survival advantages against killing by antimicrobial peptides, i.e. LL-37 and histones, but also enables replication under otherwise hostile conditions. In addition to LL-37 and histones also other antimicrobial peptides could have contributed to bacterial killing. For instance activation of the coagulation system leads to the generation of fibrinogen-derived peptides with a broad antimicrobial activity (Pahlman et al., 2013). However, the impact of such peptides was not addressed in this study. Our results further demonstrate, that due to the delayed effect of an antimicrobial attack while being challenged by second line antibiotic tetracycline, bacteria can change their proliferation profile and start to express proteins protecting them from the innate immune response. Thus, the bacteria could utilize this advantage to become more virulent and adapt their metabolism to survive in an immune primed environment. To adjust to these conditions some streptococcal serotypes have a high price to pay. For instance, proliferation of streptococci of the M1 serotype within the proteinous shield is greatly impaired when compared to the M4 and M12 serotypes. Interestingly, the M1 serotype is one of the most common and virulent streptococcal strains. Thus, it seems tempting to speculate that the loss of the temporary fitness, when covered with plasma proteins, might be of advantage to adapt to the new environmental conditions. The other two streptococcal serotypes tested, i.e. M4 and M12, have probably found mechanisms to adapt better to the host environment, as their survival in the shield is not affected to the same extent. Thus, it appears likely that the M1 strain, used in our experiments, would also adapt over time and would be able to proliferate with a similar or even higher efficiency inside the proteinous layer as the M4 and M12 strains used in this study. The simultaneous application of tetracycline with CAP-treated buffer, has a counteractive effect since this treatment leads to an inactivation of the antibiotic. The data therefore suggest that a combined administration has to be coordinated in conjunction with CAP treatment, so that it does not interfere with tetracycline administration. Whether or not this applies to other antibiotics is a limitation of this study that needs to be addressed in future experiments.

Together our experiments suggest that bacteria of the species *S. pyogenes* have developed unique mechanisms enabling them to counteract the host defense system and help to find niches, where the bacteria prepare for attacks by the innate immune system.

## Data Availability Statement

The raw data supporting the conclusions of this article will be made available by the authors, without undue reservation.

## Ethics Statement

The studies involving human participants were reviewed and approved by IRB approved protocol Dnr 2017/728. The patients/participants provided their written informed consent to participate in this study.

## Author Contributions

HJ performed conceptualization, supervision, project administration and investigation, and writing the manuscript. SP performed survival assays and RT-qPCR. KB performed fibrin clot assay. HH was involved in funding acquisition, supervision, and writing, reviewing, and editing the manuscript. All authors contributed to the article and approved the submitted version.

## Funding

This work was supported in part by the Swedish Research Council (grant no. 2019-01086). Österlund Foundation V2018/1399, and Donationerna för naturvetenskap, medicin och teknik - Medicin - 2019-09-25; Ansökan 40760. Funding for open access publication fees by Lund University Library, Fund Application 20201124.

## Conflict of Interest

The authors declare that the research was conducted in the absence of any commercial or financial relationships that could be construed as a potential conflict of interest.
