# Corrigendum: Plasma Protein Layer Concealment Protects *Streptococcus pyogenes* From Innate Immune Attack

**DOI:** 10.3389/fcimb.2021.752280

**Published:** 2021-08-24

**Authors:** Hilger Jagau, Swathi Packirisamy, Kyle Brandon, Heiko Herwald

**Affiliations:** ^1^Division of Infection Medicine, Department of Clinical Sciences, Lund University, Lund, Sweden; ^2^UCD School of Medicine, University College Dublin, Dublin, Ireland

**Keywords:** antibiotic resistance, antimicrobials, cold atmospheric plasma, hemostasis, innate immune system, *Streptococcus pyogenes*

## Figure/Table Legend

In the original article, there was a mistake in the legend for **Figure 3A**; **Figure 3C**; **Figure 4A–C**; **Figure 5A**; **Figure 5(B, C)** as published. CFU counts were used by mistake instead of the relative survival table. The correct legends appear below. The authors apologize for this error and state that this does not change the scientific conclusions of the article in any way. The original article has been updated.

**Figure 3A** Survival of the streptococcal strains AP1, AP4, AP12, MC25, *E. coli* K12 and *S. pneumoniae* F23 following incubation with buffer or plasma. The bacterial strains were grown to exponential phase and incubated in 5% human plasma and assay buffer for 30min. Bacteria were then washed and incubated for another 30min or 60min. The graph represents the relative survival rate, calculated based on the platings for the 30min and 60min time points divided through the starting CFU before the plasma and buffer incubation. Except for K12. Instead of the starting CFU after the plasma incubation, the CFU after buffer incubation was chosen. This was done since the buffer CFU differs from 112.6x10^4^ after buffer incubation to 2.6x10^4^ after plasma incubation and would mislead to a high positive survival rate by increasing to 5.7 x10^4^ at 30min. CFU counts were measured from 10^-4^ dilutions on THY agar plates. Assays were performed as technical quadruplicates with biological triplicates. Significance bars: To compare buffer and plasma sample values at the same time point a one-way ANOVA (Tukey’s multiple comparisons test) was used. *p ≤ 0.05, ***p ≤ 0.001, ns p=not significant.

**Figure 3C**. LL-37 and CTH induced bacterial killing inside a fibrin clot. S. pyogenes AP1 bacteria were grown to the exponential phase, incubated with 5% human plasma (grey dots) or buffer (black dots), and then embedded in a fibrin network. After a 30min and 60min incubation time bacterial survival was measured inside the clot. For buffer/plasma without N=18; plasma 30min CTH N=55; buffer 30min CTH N=49; plasma 60min CTH N=42; buffer 60min CTH, buffer 30min LL-37, buffer 60min LL-37, plasma 60min LL-37 all N= 40; plasma 30min LL-37 N=59 and for buffer 30min LL-37 N=60. The graphs display mean and standard error. Statistic tests performed on Prism (8.3.0) using a Mann-Whitney test. *p ≤ 0.05, ***p ≤ 0.001.

**Figure 4A–C** Survival of the streptococcal strains AP1, AP4 and AP12 upon treatment with LL-37 and CTH. The bacterial strains AP1, AP4 and AP12 were grown to exponential phase before adding to 5% human plasma or assay buffer for 30min. After a washing step the bacteria were incubated for another 30min and 60min in the presence of LL-37 and CTH (A,B,C). Incubation with buffer served as control. The graph represents the relative survival rate, calculated based on the platings for the 30min and 60min time points divided through the starting CFU before the plasma and buffer incubation. CFU counts were measured from 10^-4^ dilutions on THY agar plates. The graphs display mean and standard error. Statistic tests performed on Prism (8.3.0) using a one-way ANOVA (Tukey’s multiple comparisons test) to compare buffer and plasma sample values at the same time point and to compare similar conditions over two timepoints. ***p ≤ 0.001, **p ≤ 0.01 and ns p=not significant. Significance was calculated based on technical quadruplicates per individual biological sample with performing the assay three times independently.

**Figure 5A**. The effect of tetracycline and cold atmospheric plasma on *S. pyogenes* bacteria covered in a plasma protein layer. *S. pyogenes* AP1 bacteria were grown to the exponential phase and incubated with 5% human plasma or buffer. The bacteria were then incubated with tetracycline or in CAP-treated buffer for 30min and 60min to assay buffer. The graph represents the relative survival rate, calculated based on the platings for the 30min and 60min time points divided through the starting CFU before the plasma and buffer incubation. CFU counts were measured from 10^-4^ dilutions on THY agar plates. The graphs display mean and standard error for the 30min and 60min timepoint. Significance was calculated using Prism (8.3.0) performing one-way ANOVA (Tukey’s multiple comparisons test) based on technical quadruplicates per individual biological sample with performing the assay three times independently. *** p ≤ 0.001, ns p=not significant.

**Figure 5(B, C)**. Survival of *S. pyogenes* bacteria upon combined treatment with CAP and LL-37 or CAP and tetracycline. *S. pyogenes* AP1 bacteria were grown to the exponential phase and incubated with 5% human plasma or buffer. The bacteria were then subjected to CAP-treated buffer and added for 30min and 60min to a buffer containing LL-37 **(B)** or tetracycline **(C)**. The graph represents the relative survival rate, calculated based on the platings for the 30min and 60min time points divided through the starting CFU before the plasma and buffer incubation. CFU counts were measured from 10^-4^ dilutions on THY agar plates. The graphs display mean and standard error for the 30min and 60min time point. Significance was calculated using Prism (8.3.0) performing a one-way ANOVA (Tukey’s multiple comparisons test) based on technical quadruplicates per individual biological sample with performing the assay three times independently. *p ≤ 0.033, **p ≤ 0.01, ***p ≤ 0.001, ns p=not significant.

## Error in Figure/Table

In the original article, there was a mistake in **Figure 3A**; **Figure 4A–C**; **Figure 5A**; **Figure 5(B, C)** as published. CFU counts were used by mistake instead of the relative survival table. The corrected **Figure 3A**; **Figure 4A–C**; **Figure 5A**; **Figure 5(B, C)** are presented below. The authors apologize for this error and state that this does not change the scientific conclusions of the article in any way. The original article has been updated.

Figures, tables, and images will be published under a Creative Commons CC-BY licence and permission must be obtained for use of copyrighted material from other sources (including re-published/adapted/modified/partial figures and images from the internet). It is the responsibility of the authors to acquire the licenses, to follow any citation instructions requested by third-party rights holders, and cover any supplementary charges.

## Text Correction

In the original article, there was an error. CFU counts were by used mistake instead of the relative survival table.

A correction has been made to **Results, (1)** The protein shell promotes resistance to bacterial killing; **(2)** The protein shell delays killing from innate immunity antimicrobials**; (3)** Interference of the plasma protein shield with tetracycline and cold atmospheric plasma treatment, **(1)** 310-312 **(2)** 350-357**; (3)** 377-380:

(1)

**Figure 3A** shows that all S. pyogenes wild type strains tested were able to grow and proliferate when surrounded with a plasma protein layer.

(2)

While the preincubation with human plasma made the bacteria more resistant to the LL-37 attack with exception of the 30min time point for AP12. The protective effect was most significant in the streptococcal AP4 strain (**Figure 4B**), whereas the differences in survival of the streptococcal AP12 strain was, though statistically significant, less apparent (**Figure 4C**).

Like LL-37, also histones are considered as endogenous antimicrobial peptides. They are released for example from necrotic cells and have been shown to directly kill bacteria, fungi, and other parasites (19). As depicted in **Figure 4A–C**, when CTH were tested under the same experimental conditions, similar findings were obtained as seen for LL-37.

**Figure 3:**
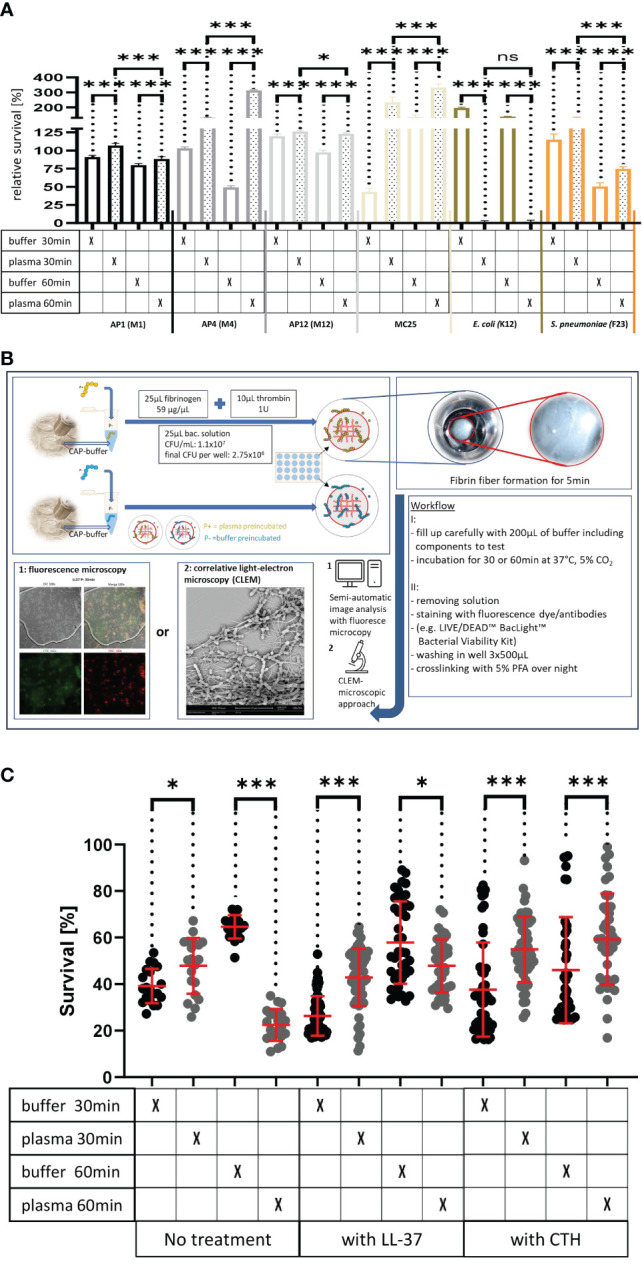
**(A)** Survival of the streptococcal strains AP1, AP4, AP12, MC25, *E. coli* K12 and *S. pneumoniae* F23 following incubation with buffer or plasma. The bacterial strains were grown to exponential phase and incubated in 5% human plasma and assay buffer for 30min. Bacteria were then washed and incubated for another 30min or 60min. The graph represents the relative survival rate, calculated based on the platings for the 30min and 60min time points divided through the starting CFU before the plasma and buffer incubation. Except for K12. Instead of the starting CFU after the plasma incubation, the CFU after buffer incubation was chosen. This was done since the buffer CFU differs from 112.6x10^4^ after buffer incubation to 2.6x10^4^ after plasma incubation and would mislead to a high positive survival rate by increasing to 5.7 x10^4^ at 30min. CFU counts were measured from 10^-4^ dilutions on THY agar plates. Assays were performed as technical quadruplicates with biological triplicates. Significance bars: To compare buffer and plasma sample values at the same time point a one-way ANOVA (Tukey’s multiple comparisons test) was used. *p ≤ 0.05, ***p ≤ 0.001, ns p=not significant. **(B)** Workflow with in vitro fibrin clot assay. Upper left starting with resuspending the buffer and plasma incubated bacteria in normal or CAP-treated buffer. Afterwards clot assay was started by mixing bacterial solution as well as fibrinogen and thrombin on the collagen coated coverslip. Initial fibrin network formation was enabled for 5min before bacterial clot was floated with buffer including components to test. 30min and 60min time points were further processed and either used for LIVE/DEAD™ BacLight™ staining or processed for correlative light-electron microscopy (CLEM). For the CLEM only scanning electron microscopy (SEM) was used to visualize fibrin network formation. **(C)** LL-37 and CTH induced bacterial killing inside a fibrin clot. S. pyogenes AP1 bacteria were grown to the exponential phase, incubated with 5% human plasma (grey dots) or buffer (black dots), and then embedded in a fibrin network. After a 30min and 60min incubation time bacterial survival was measured inside the clot. For buffer/plasma without N=18; plasma 30min CTH N=55; buffer 30min CTH N=49; plasma 60min CTH N=42; buffer 60min CTH, buffer 30min LL-37, buffer 60min LL-37, plasma 60min LL-37 all N= 40; plasma 30min LL-37 N=59 and for buffer 30min LL-37 N=60. The graphs display meanand standard error. Statistic tests performed on Prism (8.3.0) using a Mann-Whitney test. *p ≤ 0.05, ***p ≤ 0.001.

**Figure 4:**
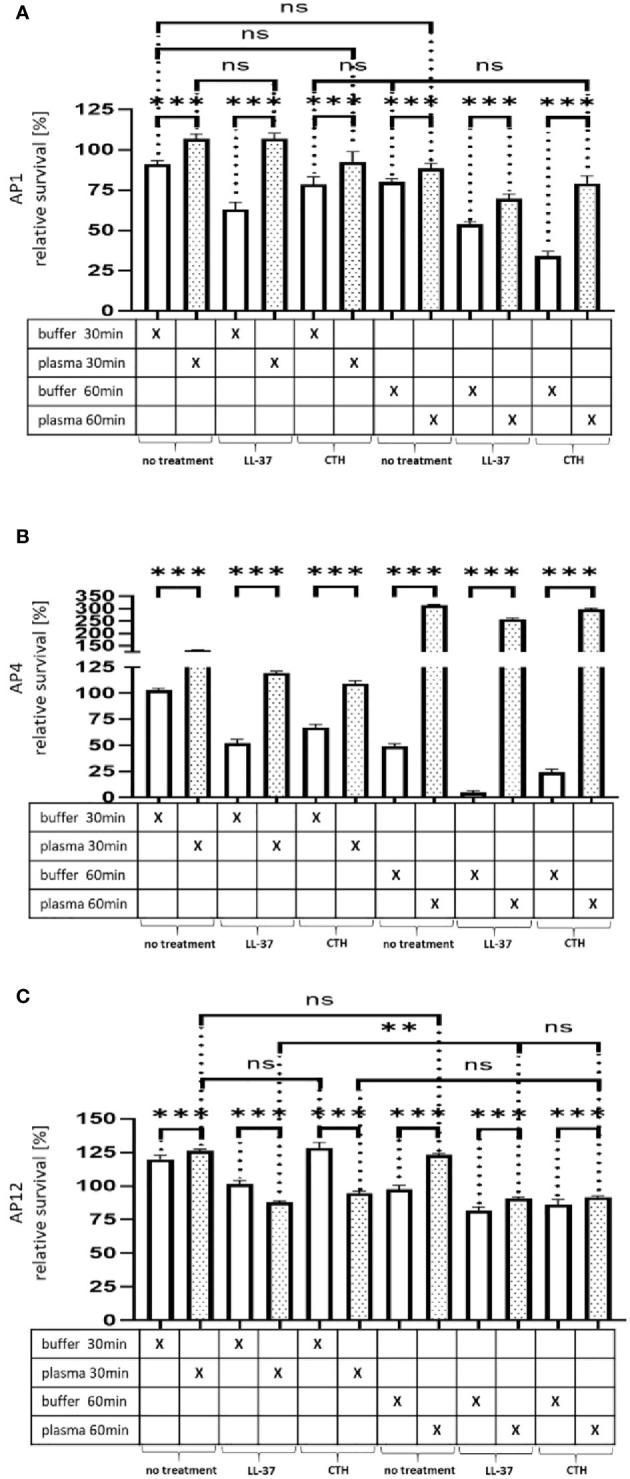
Survival of the streptococcal strains AP1, AP4 and AP12 upon treatment with LL-37 and CTH. The bacterial strains AP1, AP4 and AP12 were grown to exponential phase before adding to 5% human plasma or assay buffer for 30min. After a washing step the bacteria were incubated for another 30min and 60min in the presence of LL-37 and CTH **(A–C)**. Incubation with buffer served as control. The graph represents the relative survival rate, calculated based on the platings for the 30min and 60min time points divided through the starting CFU before the plasma and buffer incubation. CFU counts were measured from 10^-4^ dilutions on THY agar plates. The graphs display mean and standard error. Statistic tests performed on Prism (8.3.0) using a one-way ANOVA (Tukey’s multiple comparisons test) to compare buffer and plasma sample values at the same time point and to compare similar conditions over two timepoints. ***p ≤ 0.001, **p ≤ 0.01 and ns p=not significant. Significance was calculated based on technical quadruplicates per individual biological sample with performing the assay three times independently.

**Figure 5:**
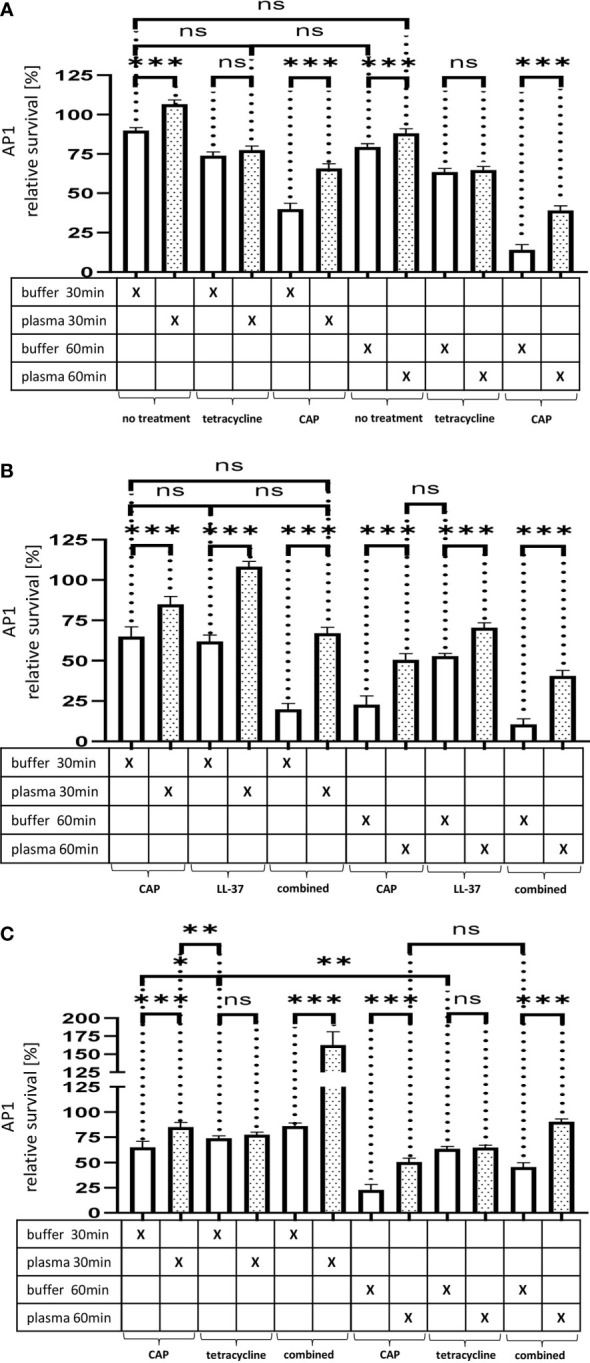
**(A)** The effect of tetracycline and cold atmospheric plasma on *S. pyogenes* bacteria covered in a plasma protein layer. *S. pyogenes* AP1 bacteria were grown to the exponential phase and incubated with 5% human plasma or buffer. The bacteria were then incubated with tetracycline or in CAP-treated buffer for 30min and 60min to assay buffer. The graph represents the relative survival rate, calculated based on the platings for the 30min and 60min time points divided through the starting CFU before the plasma and buffer incubation. CFU counts were measured from 10^-4^ dilutions on THY agar plates. The graphs display mean and standard error for the 30min and 60min timepoint. Significance was calculated using Prism (8.3.0) performing one-way ANOVA (Tukey’s multiple comparisons test) based on technical quadruplicates per individual biological sample with performing the assay three times independently. *** p ≤ 0.001, ns p=not significant. Survival of *S. pyogenes* bacteria upon combined treatment with CAP and LL-37 or CAP and tetracycline. *S. pyogenes* AP1 bacteria were grown to the exponential phase and incubated with 5% human plasma or buffer. The bacteria were then subjected to CAP-treated buffer and added for 30min and 60min to a buffer containing LL-37 **(B)** or tetracycline **(C)**. The graph represents the relative survival rate, calculated based on the platings for the 30min and 60min time points divided through the starting CFU before the plasma and buffer incubation. CFU counts were measured from 10^-4^ dilutions on THY agar plates. The graphs display mean and standard error for the 30min and 60min time point. Significance was calculated using Prism (8.3.0) performing a one-way ANOVA (Tukey’s multiple comparisons test) based on technical quadruplicates per individual biological sample with performing the assay three times independently. *p ≤ 0.033, **p ≤ 0.01, ***p ≤ 0.001, ns p=not significant.

(3)

Thus, no significant differences in streptococcal proliferation were noted under these experimental conditions. In search for alternatives to traditional antibiotic treatment, cold atmospheric plasma (CAP) has recently attracted considerable attention (20).

The authors apologize for this error and state that this does not change the scientific conclusions of the article in any way. The original article has been updated.

## Publisher’s Note

All claims expressed in this article are solely those of the authors and do not necessarily represent those of their affiliated organizations, or those of the publisher, the editors and the reviewers. Any product that may be evaluated in this article, or claim that may be made by its manufacturer, is not guaranteed or endorsed by the publisher.

