# Supplementary material for: Plasma Protein Layer Concealment Protects Streptococcus pyogenes From Innate Immune Attack
Source: Front Cell Infect Microbiol. 2021 May 20;11:633394. doi: 10.3389/fcimb.2021.633394 (PMC8173628; doi:10.3389/fcimb.2021.633394)
Supplement: Supplementary file 1 [file DataSheet_1.docx]

Supplementary Material

# Supplementary Data

# Supplementary Figures and Tables

**The supplementary figures:**

**Table S1: PCR reaction mix components for 384 well plate. FW: forward primer, RW: reverse primer**

| **Components** | **Volume per reaction**  **for 384 well plate** |
| --- | --- |
| TaqMan fast advanced master mix (2x) | 5.0μl |
| Nuclease free water | 0.5μl |
| FW primer (0.1nM) | 0.5μl |
| RW primer (0.1nM) | 0.5μl |
| Probe | 0.5μl |
| cDNA (300ng) | 3μl |
| **Total volume** | **10μl** |

Table S2: Survival counts of the clot assay (excluded counts marked in red)

| plasma  30min | buffer  30min | plasma  60min | buffer  60min | plasma  30min CTH | buffer  30min CTH | plasma  60min  CTH | buffer  60min  CTH | plasma  30min  LL-37 | buffer  30min  LL-37 | plasma  60min  LL-37 | buffer  60min  LL-37 |
| --- | --- | --- | --- | --- | --- | --- | --- | --- | --- | --- | --- |
| 58,28 | 37,71 | 10,96 | 60,29 | 57,51 | 57,12 | 84,98 | 90,59 | 31,47 | 39,19 | 45,27 | 51,81 |
| 50,77 | 47,55 | 13,10 | 63,54 | 44,01 | 77,99 | 73,33 | 63,08 | 39,5 | 42,6 | 30,88 | 51,22 |
| 58,06 | 34,38 | 24,30 | 51,35 | 51,77 | 80,5 | 72,09 | 94,49 | 13,28 | 52,9 | 45,23 | 53,45 |
| 55,54 | 38,54 | 17,61 | 71,99 | 52,88 | 72,81 | 70,47 | 95,15 | 38,08 | 41,06 | 38,02 | 45,4 |
| 57,35 | 49,59 | 34,98 | 58,40 | 57,72 | 80,72 | 98,96 | 84,94 | 53,46 | 40,66 | 42,65 | 34,89 |
| 50,27 | 53,48 | 18,74 | 63,81 | 60,53 | 78,53 | 78,16 | 85,13 | 25,48 | 44,96 | 46,98 | 38,66 |
| 62,55 | 49,24 | 27,39 | 64,10 | 49,82 | 73,75 | 60,5 | 94,73 | 62,03 | 31,64 | 29,58 | 44,51 |
| 58,10 | 46,21 | 18,51 | 67,22 | 93,11 | 82,58 | 94,23 | 56,1 | 31,86 | 35 | 55,17 | 48,91 |
| 56,92 | 30,80 | 24,26 | 62,16 | 75,76 | 46,73 | 90,42 | 61,75 | 46,95 | 31,27 | 51,96 | 50,11 |
| 67,17 | 34,35 | 32,45 | 66,75 | 58,21 | 37,06 | 86,21 | 65,14 | 55,64 | 19,48 | 39,21 | 42,48 |
| 35,28 | 42,89 | 23,79 | 59,67 | 56,03 | 44,7 | 100,68 | 66,91 | 64,64 | 25,63 | 38,04 | 45,73 |
| 50,54 | 35,93 | 20,33 | 67,97 | 61,8 | 31,8 | 95,67 | 52,67 | 47,8 | 20,27 | 32,39 | 33,48 |
| 48,28 | 45,32 | 31,94 | 71,46 | 68,96 | 47,28 | 101,73 | 30,09 | 54,1 | 25,31 | 51,2 | 44,34 |
| 40,74 | 32,83 | 12,39 | 62,61 | 77,98 | 34,99 | 38,36 | 57,62 | 50,08 | 19,06 | 34,92 | 53,85 |
| 31,53 | 30,74 | 21,61 | 65,15 | 55,14 | 39,5 | 78,29 | 37,1 | 57,41 | 19,68 | 38,67 | 32,7 |
| 25,87 | 40,06 | 26,91 | 62,10 | 56,39 | 39,01 | 58,63 | 41,8 | 45,39 | 38,04 | 37,58 | 37,63 |
| 33,34 | 32,13 | 16,68 | 69,01 | 55,92 | 32,85 | 64,25 | 44,76 | 51,58 | 20,71 | 33,11 | 42,1 |
| 45,50 | 35,85 | 27,13 | 66,95 | 66,36 | 32,65 | 55,73 | 50,86 | 54,04 | 20,88 | 38,53 | 34,63 |
|  |  |  |  | 65,21 | 22,93 | 59,57 | 38,13 | 53,47 | 17,79 | 33,06 | 36,92 |
|  |  |  |  | 49,67 | 36,07 | 59,33 | 43,98 | 22,13 | 24,98 | 36,9 | 48,12 |
|  |  |  |  | 67,07 | 25,97 | 59,06 | 28,27 | 35,54 | 23,07 | 50,82 | 35,12 |
|  |  |  |  | 70,51 | 62,09 | 63,06 | 30 | 49,64 | 31,81 | 62,51 | 59,87 |
|  |  |  |  | 50,37 | 35,4 | 73,36 | 24,7 | 56,38 | 26,11 | 60,97 | 69,09 |
|  |  |  |  | 47,8 | 22,49 | 73,02 | 24,67 | 51,37 | 39,73 | 55,28 | 63,81 |
|  |  |  |  | 49,37 | 33,62 | 53,98 | 31,82 | 38,3 | 18,59 | 45,93 | 53,7 |
|  |  |  |  | 59,17 | 64,23 | 63,11 | 29,79 | 43,01 | 22,12 | 59,35 | 73,58 |
|  |  |  |  | 70,07 | 45,15 | 53,88 | 24,64 | 32,84 | 20,29 | 64,14 | 77,86 |
|  |  |  |  | 66,31 | 27,37 | 37,3 | 34,59 | 41,35 | 31,93 | 61,93 | 77,08 |
|  |  |  |  | 67,46 | 34,7 | 38,28 | 24,98 | 30,67 | 32,63 | 71,87 | 78,39 |
|  |  |  |  | 70,91 | 16,41 | 41,92 | 28,31 | 16,76 | 20,8 | 39,89 | 83,93 |
|  |  |  |  | 63,04 | 19,87 | 67,79 | 26,27 | 21,14 | 42,29 | 70,65 | 80,23 |
|  |  |  |  | 70,77 | 16,22 | 65,77 | 32,3 | 40,95 | 26,42 | 49,28 | 59,49 |
|  |  |  |  | 68,25 | 32,58 | 40,93 | 25,78 | 37,01 | 20,6 | 55,83 | 83,3 |
|  |  |  |  | 68,41 | 23,67 | 42,72 | 30,61 | 33,95 | 18,06 | 52,42 | 81,6 |
|  |  |  |  | 81,13 | 20,34 | 42,07 | 35,52 | 46,26 | 27,48 | 55,94 | 74,32 |
|  |  |  |  | 39,59 | 31,94 | 48,45 | 32,77 | 43,94 | 27,13 | 53,22 | 72,53 |
|  |  |  |  | 27,38 | 20,96 | 25,1 | 27,79 | 41 | 29,44 | 57,56 | 71,36 |
|  |  |  |  | 38,93 | 20,44 | 37,67 | 42,97 | 47,75 | 30,43 | 59,29 | 87,8 |
|  |  |  |  | 32,13 | 22,81 | 57,37 | 24,85 | 51,54 | 19,38 | 35,05 | 89,02 |
|  |  |  |  | 39,1 | 35,2 | 33,26 | 25,28 | 11,32 | 28,47 | 54,41 | 70,99 |
|  |  |  |  | 35,19 | 20,1 | 16,9 |  | 18,5 | 27,11 |  |  |
|  |  |  |  | 46,52 | 23,68 | 55,65 |  | 41,17 | 19,91 |  |  |
|  |  |  |  | 46,44 | 16,94 | 40,42 |  | 38,18 | 18,22 |  |  |
|  |  |  |  | 52,18 | 16,22 | 41,03 |  | 35,02 | 19,05 |  |  |
|  |  |  |  | 48,46 | 17,14 |  |  | 39,42 | 18,61 |  |  |
|  |  |  |  | 53,32 | 23,85 |  |  | 51,38 | 19,85 |  |  |
|  |  |  |  | 48,29 | 20,46 |  |  | 42,27 | 20,72 |  |  |
|  |  |  |  | 39,01 | 18,08 |  |  | 44,24 | 16,89 |  |  |
|  |  |  |  | 41,71 | 26,64 |  |  | 48,81 | 17,28 |  |  |
|  |  |  |  | 35,62 |  |  |  | 52,64 | 17,73 |  |  |
|  |  |  |  | 53,68 |  |  |  | 42,03 | 20,93 |  |  |
|  |  |  |  | 50,01 |  |  |  | 49,91 | 17,22 |  |  |
|  |  |  |  | 32,26 |  |  |  | 54,83 | 30,7 |  |  |
|  |  |  |  | 25,69 |  |  |  | 31,45 | 18 |  |  |
|  |  |  |  | 50,59 |  |  |  | 59,94 | 23,46 |  |  |
|  |  |  |  |  |  |  |  | 47,11 | 20,91 |  |  |
|  |  |  |  |  |  |  |  | 50,6 | 24,41 |  |  |
|  |  |  |  |  |  |  |  | 62,39 | 22,05 |  |  |
|  |  |  |  |  |  |  |  | 53,02 | 34,98 |  |  |
|  |  |  |  |  |  |  |  |  | 20,96 |  |  |
|  |  |  |  |  |  |  |  |  |  |  |  |

## Figure S1: Pre-incubation in 5% human plasma influence the expression of virulence and metabolic genes of AP1 when challenged with tetracycline (tet) and CAP. (A) Ct-values at 0min after treatment, (B) Ct-values at 30min after treatment, (C) Ct-values at 60min after treatment. AP1 grown to exponential phase were incubated in solutions of 5% human plasma (PL) or buffer (B) and exposed to buffers treated with tetracycline (30μg/ml) and CAP (30s, 2.5bar, 3L/min) 0min (0’), 30min (30’) and 60min (60’). RNA was extracted using the RiboPure™ RNA Purification Kit and cDNA was synthesized with QuantiTect Reverse Transcription Kit, Qiagen. Real time qPCR was run in QuantStudio™ 7 Flex Real-Time PCR Instrument, 384-well from Thermo Fisher. Mean Ct (mCt) values of the replicates are plotted.
